# Introduction to the Special Issue on Pediatric Neuro-Oncology

**DOI:** 10.3390/bioengineering5040109

**Published:** 2018-12-11

**Authors:** Natasha Pillay Smiley, Soumen Khatua

**Affiliations:** 1 Department of Hematology/Oncology/SCT. Ann & Robert H. Lurie Children’s Hospital, Chicago, IL 60611, USA; 2 Department of Pediatrics, The University of Texas MD Anderson Cancer Center, Houston, TX 77030, USA; skhatua@mdanderson.org

**Keywords:** brain tumor, pediatrics, advancements, molecular biology

Pediatric Neuro-Oncology is a highly specialized field encompassing molecular biology, clinical acumen, evidence based medicine, cancer genetics and neuropsychological care for the diagnosis and treatment of children with central nervous system (CNS) tumors. In data acquired by the National Institute of Health’s (NIH) Surveillance, Epidemiology and End Results (SEER) Program, there were 3.1 new cases of childhood brain tumors per 100,000 people from 2011–2015. This represents the second most common pediatric cancer diagnosis (17.2% overall, second only to leukemia) as well as the leading cause of mortality [1]. This special edition of the *Bioengineering* Journal highlights major advancements in pediatric neuro-oncology as well as our current challenges. 

The SEER data describes the overall survival of pediatric CNS tumors to be approximately 70%, an improvement from less than 60% from the 1970s [1]. This statistic can be misrepresentative as pediatric CNS tumors are heterogenous with diverse survival outcomes, ranging from the mostly indolent nature of low grade gliomas with a 20 year overall survival (OS) of 80% [2] to the highly aggressive diffuse intrinsic pontine glioma (DIPG) with a 2 year OS of less than 10%. [3,4]. 

Although survival has not dramatically increased for DIPG and high grade glioma over the last few decades, there has been an increase in the survivability of other tumors such as the medulloblastoma (MB) and atypical teratoid rhabdoid tumor (ATRT). This has resulted from the surge of genomic and epigenomic data of pediatric brain tumors- forging an era of biologically targeted therapy with improved survival outcome of CNS tumors. This is best illustrated by the story of medulloblastoma subtyping and the somewhat recent discovery of ATRT. We now have established molecular subgroups with defined demographics, oncogenic drivers and risk stratification based treatment strategies. Retrospective analysis of medulloblastoma patients have determined that children with WNT subgrouping have a significantly higher overall survival, and thus, clinical trials are now focused on changing upfront treatment of these children to mitigate the profound late effects medulloblastoma patients face [5]. 

Atypical Teratoid Rhabdoid tumor (ATRT) was only described in the 1980s, previously thought to be a type of medulloblastoma or supratentorial PNET [6,7]. Advances in histologic characterization and FISH for chromosome 22 helped to classify this as a separate entity. ATRT is a disease of primarily infants, and was nearly always fatal, with the 3 years survival of children treated with the Pediatric Oncology Group (POG) infant studies of less than 10% [7]. However, treatment with high dose chemotherapy and/or autologous transplantation and radiation has now led to improved survival in this population of young children. The two years overall survival for the DFCI regimen is 70 +/− 10%, using intrathecal chemotherapy, focal or craniospinal radiation and dose intensive chemotherapy [7]. The Vienna regimen, which had a smaller cohort of patients, is also a dose intensive regimen and has an excellent 5 years overall survival of 100% using methotrexate, intrathecal chemotherapy, anthracyclines, focal radiation and autologous transplantation [8]. As expected, there are long term effects from these treatments and children have been found to have neurocognitive sequelae even in the absence of radiation [9]. Molecular subtyping using methylation profiles has now delineated three subtypes of ATRT, with the hope that risk stratification can help further improve survival while decreasing toxicity and long term effects [10].

Arguably, as illustrated above, the most critical advancement in our field is the attainment of an accurate diagnosis, which has implications not only for individual patient care but also for basic science and clinical trial research [11]. The World Health Organization (WHO) Classification of CNS Tumors represents a consensus opinion from world experts and allows pathologists and neuro-oncologists across the world the opportunity to have guidelines to define CNS tumors [12]. A major change occurred in the most recent edition of the guidelines set forth in 2016. Distinct molecular characteristics were integrated into the classification of CNS tumors, allowing for an “integrated diagnosis” that is “layered” with both histologic features and molecular biology [12]. Histologic analysis depends on defining tumors by cell of origin and level of differentiation. This is accomplished by examining “hematoxylin and eosin-stained (H & E) sections, immunohistochemical expression of lineage associated proteins and ultrastructural characterization” [11]. This well-established method is now augmented by molecular analysis of the genotype of these tumors. This change brings scientific advancement into direct patient care and is an example of the innovative nature of this field. Less formally, but perhaps no less important, parameters such as neuro-imaging and clinical course is also taken into account to complete the integrated diagnosis. 

Translational research bridges the gap between basic science and cancer treatments. Major advancements in next generation sequencing technology has led to greater understanding of cancer genomes and thus led to potential cures for patients [13]. This is beautifully illustrated in the landscape of pediatric low grade glioma, the most common central nervous system tumor in children [13]. The “integrated diagnosis” now routinely includes histologic grading as well as whether the tumor has particular aberrations in the MAP kinase (MAPK) pathway [13,14,15,16]. While multiple genetic changes have been seen in pediatric low grade glioma, the most common involve the MAPK pathway, specifically, either an activating point mutation of BRAFV600E or activating of BRAF through a tandem duplication. This results in the KIAA 1549-BRAF fusion protein [14,15,16]. Molecular analysis has been correlated with histologic characterization, and 70-90% of pilocytic astrocytomas have been found to have a BRAF-KIAA1549 fusion [15]. In addition, BRAF v600 E has been found to be aberrant in other low grade gliomas such as pilomyxoid astrocytomas [14]. Drugs have been developed to selectively inhibit these targets, and early phase clinical trials have been undertaken to understand their tolerability and efficacy in children. The traditional methods of treating low grade glioma are systemic chemotherapy and, more remotely, radiation. These modalities can cause significant late effects in patients, and maximizing efficacy while minimizing long term effects are important in a population with an expected long term survival [2,15]. A Phase I trial through the Pediatric Brain Tumor Consortium (PBTC) used selumetinib (AZD6244, AstraZeneca), an oral small molecule inhibitor of MEK-1/2, in children with recurrent low grade glioma. A dose was established to perform the phase II trial, in which efficacy will be tested. However, promising antitumor effect was seen in the phase I trial [16]. 

In this special edition of *Bioengineering*, we hope to demonstrate the wide breath of science and medicine that occurs in the field of pediatric neuro-oncology (Table 1). Faced with substantial mortality in children with aggressive tumors as well as significant morbidity of survivors, we are always challenged to learn more about these disease entities and improve the outcomes of these children. Topics that will be discussed further in this edition are: molecular biology in pediatric gliomas, clinical relevance of preclinical models, update on radiation therapy for pediatric CNS tumors, molecular neuro-imaging, embryonal tumors and targeted therapeutics, neurocognitive and psychosocial outcomes and palliative care in children with central nervous system malignancies. 

## Figures and Tables

**Table 1:** Published papers in Special Issue Update in Pediatric Neuro-Oncology.

Papers	Reference
Preclinical Models of Pediatric Brain Tumors—Forging Ahead	[17]
Cutting Edge Therapeutic Insights Derived from Molecular Biology of Pediatric High-Grade Glioma and Diffuse Intrinsic Pontine Glioma (DIPG)	[18]
Polo-Like Kinase 4 (PLK4) Is Overexpressed in Central Nervous System Neuroblastoma (CNS-NB)	[19]
Embryonal Tumors of the Central Nervous System in Children: The Era of Targeted Therapeutics	[20]
Radiotherapy Advances in Pediatric Neuro-Oncology	[21]
The Emerging Role of Amino Acid PET in Neuro-Oncology	[22]
Palliative Care for Children with Central Nervous System Malignancies	[23]
Neurocognitive and Psychosocial Outcomes in Pediatric Brain Tumor Survivors	[24]
